# Carcinoma of the Uterus Complicated with Universal Dropsy

**Published:** 1855-08

**Authors:** H. Ritchie

**Affiliations:** Chicago


ARTICLE II.—*Carcinoma of the Uterus complicated with universal Dropsy.* By H. Ritchie, M. D., of Chicago.

Mrs. D., aged 32 years, was first seen as a patient last November.

*History.*—For eighteen months previous has had continued uterine hemorrhage, during which time she became pregnant, and was delivered at her full period, of a living child. Her appearance at this time was very anœmic, with œdematous extremities. She still had a sanguineous discharge from vagina, unaccompanied with pain or fœtor. The lungs were apparently healthy, and nothing abnormal about the heart was detected at that time; but subsequently, the second sound of the heart was modified from the short, quick sound, into a prolonged clanking sound, or a ringing one; no murmurs indicative of valvular disease could be distinguished. On vaginal examination, a large circular indurated mass was felt, evidently the Cervix Uteri; projecting from the indurated and patulous os, were several irregular indurated growths, and filling the os, producing from the weight of the diseased mass partial prolapsus.

*Treatment.*—Ferruginous medicines and occasional hydragogue cathartics were at first administered; but these remedies availed but little, though persevered in for two months, the hemorrhage still continued, and the œdema became universal, confining her to her bed.

There was desire for food, but upon taking it she complained of a feeling of distension in the epigastric region, of so unpleasant a nature as to deter her from gratifying her desire.

This condition, gradually becoming aggravated, continued for several months, until ascities became the prominent dropsical condition, to such an extent as to interfere with respiration; and as hydragogue cathartics and other remedies could no longer be tolerated, paracentesis abdominis was resorted to, and 3½ gal. of serum, drawn off, relieving the dyspnœa; but re-accumulation was again established, and progressed rapidly.

During the accumulation of fluid in the peritoneum, the œdema of the extremities partially subsided, and large hemorrhagic spots appeared on both lower extremities.

The above case was under the treatment of a medical friend, and as it was a remarkable instance of complicated disease, the writer had repeated opportunities of examining it, for it continued for a period of nearly eighteen months under treatment, when within a few days past it terminated in death.

*Post Mortem.*

Lungs collapsed, cavity of pleura filled with fluid, the pericardium also distended with it. The walls of the right ventricle and auricle of the heart very much attenuated, and cavities filled with coagula, valves normal, left ventricle hypertrophied with auricle, and valves healthy.

Before examining abdomen, drew off a gallon of fluid, liver, kidneys and bowels apparently healthy, a few abnormal adhesions of old date. Pelvis;—Bladder collapsed and healthy, the broad ligament of the uterus on right side perforated, and encephaloid matter protruding through it near the connection of bladder and uterine walls, appearing on first view as though the bladder was involved, but on passing the sound found no appertμre in bladder, but upon its reflection from the symphyses, the anterior wall of the vagina was found perforated, and cancerous growths projecting through it. Indeed, all the uterus, except the fundus, was a mass of fungoid disease. The iliac veins below their bifurcation were plugged, apparently with the same, very much enlarged, and their coats attenuated ; above the bifurcation, as far as traced, the cava was patulous.

*Remarks.*—Here was a clear revelation of encephaloid disease of the uterus ; but this is not sufficient to account for ascites, and universal œdema, except so far as the continued hemorrhage kept up the æncmic condition ; the plugging of the iliac veins was sufficient to account for the dropsical condition of the lower extremities, but there was no pathological condition discovered in the abdomen to elucidate the cause of ascites. We must, therefore attribute the latter and the œdema of the upper extremities, and probably the primary cause of plugging of the iliac veins, to the condition of the right side of the heart, the ventricle performing its function feebly, and having as a consequence an accumulated column of venous blood pressing upon its attenuated walls.

